# Long-Term Outcomes of COVID-19 in Hospitalized Type 2 Diabetes Mellitus Patients

**DOI:** 10.3390/biomedicines12020467

**Published:** 2024-02-19

**Authors:** Zemfira Khamidullina, Diana Avzaletdinova, Diana Gareeva, Tatyana Morugova, Irina Lakman, Kristen Kopp, Lukas Fiedler, Lukas J. Motloch, Naufal Zagidullin

**Affiliations:** 1 Department of Endocrinology, Bashkir State Medical University, Lenin Str. 3, 450008 Ufa, Russia; khamidullina.zemfira@mail.ru (Z.K.); hyppocrat@mail.ru (D.A.); tmorugova@yandex.ru (T.M.); 2 Department of Internal Medicine, Bashkir State Medical University, Lenin Str. 3, 450008 Ufa, Russia; danika09@mail.ru; 3 Laboratory for the Study of Socio-Economic Problems of the Regions, Ufa University of Science and Technology, Z. Validi Str. 32, 450076 Ufa, Russia; lackmania@mail.ru; 4 Clinic for Internal Medicine II, Paracelsus Medical University, Muellner Hauptstrasse 48, 5020 Salzburg, Austria; k.kopp@salk.at (K.K.); lukas.motloch@ooeg.at (L.J.M.); 5 Department of Internal Medicine, Cardiology, Nephrology and Intensive Care Medicine, Hospital Wiener Neustadt, 2700 Wiener Neustadt, Austria; flukas@me.com; 6 Department of Internal Medicine II, Salzkammergut Klinikum, OÖG, 4840 Voecklabruck, Austria; 7 Department of Cardiology, Kepler University Hospital, Medical Faculty, Johannes Kepler University, 4040 Linz, Austria

**Keywords:** COVID-19, diabetes mellitus, outcomes, risk factors, mortality, follow-up

## Abstract

With the onset of the coronavirus pandemic, it has become clear that patients with diabetes are at risk for more severe and fatal COVID-19. Type 2 diabetes mellitus (T2D) is a major risk factor for adverse COVID-19 outcomes. The goal of study was to assess the characteristics and outcomes of hospitalized patients with COVID-19 with or without T2D in the hospital and at 10-month follow-up (FU). Methods: A total of 2486 hospitalized patients in the first wave of COVID-19 were analyzed according to the absence/presence of T2D, with 2082 (84.1%) patients in the control COVID-19 group and 381 (15.5%) in the T2D group. Twenty-three patients had other types of diabetes and were therefore excluded from the study. In-hospital mortality and cardiovascular endpoints (myocardial infarction, stroke, cardiovascular deaths and hospitalizations and composite endpoints) at the 10-month follow-up were analyzed. To remove bias in patients’ characteristics disproportion, Propensity Score Matching (PSM) was used for hospital and follow-up endpoints. Results. Hospital mortality was considerably greater in T2D than in the control COVID-19 group (13.89% vs. 4.89%, *p* < 0.0001), and the difference remained after PSM (*p* < 0.0001). Higher glucose-level T2D patients had a higher mortality rate (*p* = 0.018). The most significant predictors of hospital death in T2D patients were a high CRP, glucose, neutrophils count, and Charlson Comorbidity Index. The follow-up of patients over 10 months showed a non-significant increase for all endpoints in the T2D group (*p* > 0.05), and significant increase in stroke (*p* < 0.042). After the PSM, the difference decreased in stroke (*p* = 0.090), but became significant in cardiovascular hospitalizations (*p* = 0.023). Conclusion. In T2D patients with COVID-19, an increase in hospital mortality, stroke and cardiovascular hospitalizations rates in the follow-up was observed.

## 1. Introduction

Since December 2019, more than 0.6 billion persons have been infected with the severe acute respiratory syndrome coronavirus 2 (SARS-CoV-2), and more than 6 million died among them. The collision between the two global pandemics of coronavirus disease 2019 COVID-19 and type 2 diabetes (T2D) has led to the grim reality that T2D is already the second most common comorbidity of COVID-19 [1]. With regard to the current COVID-19 pandemic, several recent studies, though with limited participants, have already suggested that T2D is a common comorbidity and constitutes a higher proportion of patients with severe and ICU-admitted cases of COVID-19 than patients with mild symptoms [1,2].

Diabetes mellitus (DM) is emerging as a critical risk factor for poor prognosis of COVID-19, with a recent meta-analysis reporting that COVID-19 patients with pre-existing DM have a 3-fold increased risk of in-hospital mortality [3,4,5,6,7]. One population-based cohort study in England showed the increased risk of COVID-19-related death in people with diabetes and obesity [8]. These findings are in line with the available literature on the adverse prognostic impact of diabetes on other viral infections, including influenza [9]. Diabetes worsens the outcome of virtually any acute or chronic medical condition, resulting in a shortened life expectancy. It was found that blood glucose levels are emerging as a critical prognostic factor for COVID-19 mortality in both patients with and without DM [10,11,12].

As the COVID-19 pandemic has progressed, there has been a growing awareness of the long-term impacts of the COVID-19 infection. The proposed pathophysiology involves direct viral toxicity, microvascular and endothelial damage, immune system dysregulation, hypercoagulable state, and changes in the angiotensin-converting enzyme pathway, in addition to immediate sequelae of hospitalization with COVID-19. Understanding the post-COVID syndrome is crucial for multidisciplinary treatment and rehabilitation.

The goal of the study was to assess the outcomes of hospitalized patients with COVID-19 with or without T2D in hospital and at 10-month follow-up (FU).

## 2. Materials and Methods

### Study Cohort, Data Collection and Analyses

The study was performed in accordance with the standards of good clinical practice and the principles of the Declaration of Helsinki, receiving approval by the ethics commission of the Bashkir State Medical University (N11, 2020). All the patients signed informed consent.

**Figure 1:**
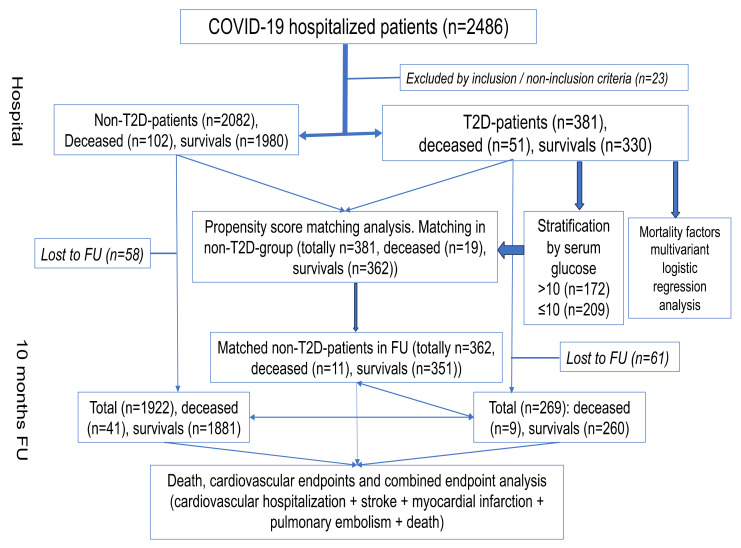
Design of the study.

A total of 2486 patients (Figure 1) were hospitalized due to COVID-19 (Bashkir State Medical University Hospital, Bashkir State, Russian Federation) between 1 January 2020, and 31 January 2021, during the first COVID-19 wave (Alpha) and retrospectively studied. Inclusion criteria for the control COVID-19 group were hospitalization in a dedicated COVID-19 unit with COVID-19 diagnosis confirmed biologically (SARS-CoV-2 PCR test) and/or clinically/radiologically (i.e., as ground-glass opacity and/or crazy paving on chest computed tomography (CT) scan). Inclusion criteria for the study group included hospitalization in a dedicated COVID-19 unit with COVID-19 diagnosis confirmed biologically (by SARS-CoV-2 PCR test) and/or clinically/radiologically (i.e., as ground-glass opacity and/or crazy paving on chest CT scan) and medical history of T2D. Exclusion criteria for study group were other types of DM (T1D, gestational DM). Thus, we performed a retrospective study of 2486 cases of COVID-19, among which 381 had pre-existing T2D and 23 had other DM types. In total, 2463 patients were enrolled in the study.

In all patients, demographics (age and sex), concomitant cardiovascular risk factors (smoke, hypertension, and dyslipidemia), comorbidities (T2D and its duration, other types of DM, chronic obstructive pulmonary disease (COPD), and history of cancer), complications (cardiovascular disease and microangiopathy), presence or absence of COVID-19-related pneumonia or interstitial lung disease, and any ongoing therapies before hospitalization were recorded. We also collected information on symptoms of COVID-19 upon admission (time from onset of symptoms to hospitalization; and presence of weakness, aches, dyspnea at rest, and dyspnea upon exertion). Vital signs were recorded at the time of admission: systolic and diastolic blood pressure (BP), heart and respiratory rate, oxygen saturation, and PaO_2_/FiO_2_ ratio.

Survival status at 10 months was recorded using the distant data approach “ProMed” (Program for Medical Cases Monitoring), and patients were contacted by phone to assess survival status.

Statistical analysis: The cohort was divided into two groups, according to the presence or absence of diabetes. T2D was defined as diagnosed, treated diabetes on admission. Baseline demographics and clinical characteristics were expressed as median (Me) and interquartile range (Q1 and Q3) for continuous numerical variables and the frequency (percentage) for categorical variables. Between-group comparisons were performed with a Mann–Whitney–Wilcoxon test for numerical variables (Wilcoxon text for dependent variables and Mann–Whitney for independent) and a Chi-squared test (including Yates correction) for categorical variables. The threshold for statistical significance was set to *p* < 0.05. All statistical tests were two-sided and were performed with R (version 4.3.1) https://www.r-project.org/ (accessed on 1 November 2023).

Propensity Score Matching (PSM) was used to remove bias in the control and T2D groups for in-hospital mortality and FU post-discharge endpoints. First, potential confounders were identified among those variables showing significant differences between control and T2D groups (at *p* < 0.05). Next, the reliability of the choice of confounders was checked using logit regression, where the patient’s inclusion in the control group or the diabetes group was considered a target variable. Based on the obtained results of assessing the logistic regression coefficients, the Propensity Score Index (PSI) of the patient’s inclusion in each of the groups was calculated. Based on the obtained PSI values, a comparison was made using the nearest neighbor method for pairs of patients, and those patients with a high index were removed from the control group so that the groups became balanced and there were no characteristics in which the samples differed. The quality control of the PSM procedure was monitored based on the analysis of the variation ratio for the values of the confounders before and after randomization, as well as on the basis of the analysis of eQQ graphs. After the pseudo-randomization, the statistical significance in the difference in the frequency of in-hospital and post-discharge endpoints in the new balanced groups was assessed. For the PSM analysis, we used the “MatchIt” library in R.

To identify in-hospital mortality risk factors in patients with T2D, multivariate logistic regression was used, the coefficients of which were estimated using the maximum likelihood method. The univariate regression model was first constructed for each variable, and coefficients with *p* < 0.05 were selected. For a better interpretation of the modeling results based on the calculation of odds ratios, significant risk predictors were preliminarily binarized, that is, converted into nominal variables according to the principle >/≤ cutoff threshold. The cutoff point for each trait was found based on an ROC analysis. The quality of the logistic regression was controlled based on the McFadden coefficient of determination (R2MF) and the significance of the regression equation as a whole (likelihood ratio (LR) test).

## 3. Results

### 3.1. Demographics and Characteristics

Baseline characteristics and laboratory parameters of 2463 hospitalized patients at the time of admission are presented in Table 1. The median age (Me [Q1, Q3]) of our study population was 59 [58; 59] years. In the T2D group (study group), the median age was higher, i.e., 66 [65; 68], than in the non-diabetic (control COVID-19) group, 57 [56; 58] years (*p* < 0.0001). The total cohort comprised 1056 men (42.9%) and 1407 women (57.1%): 126 men (33.07%) and 255 (66.93%) women in the study group, and 930 men (44.67%) and 1152 (55.33%) women in the control COVID-19 group (*p* < 0.0001). The prevalence of established DM upon hospital admission was 16.3%, and 15.5% T2D was in patients with laboratory-confirmed COVID-19, correlating with previous publications [4].

The major complaints for both groups included weakness (95.9%) and dyspnea upon rest (33.4%), similar to the general population of patients [2,13,14,15]. Patients with T2D were relatively more often admitted in severe (20.37% vs. 10.5% at *p* < 0.0001) or moderate condition (88.91% vs. 79.63% at *p* < 0.0001) compared to the control group.

The median percentage of lung damage in the T2D group was 40% [40; 44], and it was 40% [36; 40] in the control group (*p* = 0.0015). Also, patients with T2D were more often transferred to non-invasive lung ventilation (NIVL) and mechanical lung ventilation (MLV): 4.55% versus 1.35% (*p* = 0.0001) and 9.19% versus 3.9% (*p* < 0.0001), respectively. Moreover, MLV in both groups was performed more often than NIVL. The median hospital stay in the T2D group was 11 [11; 12] compared to 11 [11; 11] days in the control group (*p* = 0.0018), and the total duration of pre-admission disease (number of days from the onset of the first symptoms to hospitalization) was 16 [15; 16] and 15 [15; 15] days, respectively (*p* = 0.0008).

Pre-existing arterial hypertension (80.31% versus 36.97%, *p* = 0.0001), coronary heart disease (CHD; 22.57% versus 9.37%, *p* = 0.0001), chronic heart failure (CHF, 19.16% versus 8.46% at *p* < 0.0001), myocardial infarction (9.19% vs. 2.79% at *p* < 0.0001) and acute cerebrovascular accident (5.25% vs. 2.21% at *p* = 0.0001), valve replacement (1.05% versus 0.29% at *p* = 0.0870), obesity (4.72% versus 1.73% at *p* < 0.0001), and chronic kidney disease (15.75% versus 2.11%, *p* = 0.0001) were more frequent in the T2D group compared to the non-diabetic group. However, COPD was more often observed in the control group (1.84% versus 3.21% at *p* = 0.071).

The Charlson Comorbidity Index (CCI) was higher in patients with T2D compared with patients in the control group: 4.0 [4.0; 4.0] versus 1.0 [1.0; 1.0] (*p* < 0.0001). The Mann–Whitney test revealed significant differences in GFR in the study groups: in the group with T2D, GFR was 62 mL/min/1.73 m^2^, and in the group without diabetes, it was 71 mL/min/1.73m^2^ [70; 71] (*p* < 0.0001) upon admission. The level of urea in the T2D group was significantly higher than in the control group, 6.95 mmol/L vs. 5.39 mmol/L (*p* < 0.0001). The level of fasting venous plasma glycemia (FPG) in the T2D group was also expectedly higher: 9.46 mmol/L versus 6.18 mmol/L (*p* < 0.0001). The median C-reactive protein (CRP) in the T2D group was 42 mg/L, and in the group without diabetes, it was significantly lower 24 mg/L (*p* < 0.0001). A comparison of the median albumin showed significant differences between the studied groups: 40.4 g/L [39.7; 41.0] in the T2D versus 40.9 g/L [40.6; 41.2] in controls, *p* = 0.016.

The tendency for the more pronounced cytokine response was observed in patients with T2D compared to patients without diabetes: interleukin-6 (IL-6) level was 8.46 pg/mL [5.08; 14.27] vs. 6.18 pg/mL [4.94; 7.89] accordingly (*p* = 0.0907). Procalcitonin, according to Mann–Whitney analysis, was significantly higher in patients with T2D, i.e., 0.14 ng/L, compared to those without T2D, i.e., 0.09 ng/L (*p* < 0.0001).

### 3.2. COVID-19 Hospital Mortality in T2D Patients

The overall inpatient mortality rate was 6.2% and was almost three times higher in T2D patients compared to controls: 13.39% versus 4.89% (*p* < 0.0001). The median age of the deceased patients was 71 [69; 74] years, and the Charlson Comorbidity Index was 6 [5; 7] points, compared to survivors with a median age of 65 [64; 67] years (*p* < 0.0001) and comorbidity index of 4 [4; 4] points (*p* < 0.0001). The biserial correlation coefficient of death from T2D patients’ age was r = 0.242 (*p* = 0.0211), and Chuprov’s contingency coefficient from Charlson Comorbidity Index and death was r = 0.328 (*p* < 0.0001). The condition upon admission of in-hospital deceased patients was marked as severe in 75% vs. 12.7% in patients surviving hospitalization and subsequently discharged (*p* < 0.0001).

To obtain a prognostic model for occurrence of a lethal outcome in severe COVID-19 in patients with T2D, we used those risk factors which significantly differed between the groups of patients who died in the hospital and discharged patients, namely age, CCI, urea, glomerular filtration rate (GFR), aspartate aminotransferase (AST), albumin, CRP, creatine kinase (CK), procalcitonin, ferritin, glucose, neutrophils, thrombocytes, and D-dimers (Supplementary Materials Table S1). Also, the rate of chronic kidney disease (CKD) differed between surviving and deceased patients (*p* = 0.0413); however, this factor was not included in the univariant model because it was already used in the Charlson Comorbidity Index calculation.

As part of a univariate logistic regression analysis, a large list of risk factors that could be associated with a firm endpoint hospital death was studied. It was necessary to establish which of the listed factors have the most significant impact on the differentiation of groups with the development of a fatal outcome in COVID-19. As a result of the univariate analysis, it was revealed that at a level of *p* < 0.05 age, patient condition, low albumin, GFR and platelets, high urea, CK, CRP, neutrophils, ferritin, glucose, procalcitonin, and Charlson Comorbidity Index were associated with hospital mortality (Supplementary Materials Table S2).

In the multivariate logistic regression, the statistically significant predictors of hospital mortality at *p* < 0.05 were the Charlson Comorbidity Index (CCI), CRP, glucose, and neutrophils. For a better interpretation of the results and calculation of odds ratios, the values of these predictors were binarized in the form of dummy-variables, where the cutoff points found when constructing ROC curves for each of the considered predictors served as the separation threshold. The cutoff point values are summarized in Table 2.

**Figure 2:**
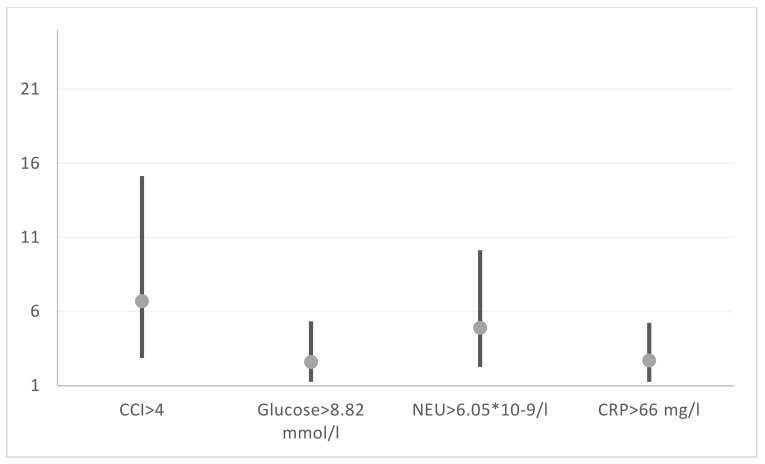
Forest plot of ORs for COVID-19-associated mortality in T2D patients. CCI—Charlson Comorbidity Index; CRP—C-reactive protein.

Multivariate logistic regression analysis with binarized risk factors. The Charlson Comorbidity Index > 4, CRP > 66 mg/L, glucose >8.82 mmol/L, and neutrophils > 6.05 × 10^9^/L (R^2^_MF_ = 0.2457, LR = 59.48, *p* < 0.001) enabled a calculation of the odds ratios (ORs) and the corresponding confidence interval (CI) with a reliability of 95% for each predictor of the risk of hospital death (Table 3, Figure 2).

T2D and non-T2D had bias in disproportions in age, gender, arterial hypertension, CHD, CKD, etc. (Table 1), and a Propensity Score Matching procedure was performed to eliminate the bias in the differences in the two groups. First, patient variables were selected as candidate confounders, and the significant differences were set at *p* < 0.05: gender (m/f), age (years), arterial hypertension (yes/no), chronic kidney disease (yes/no), coronary heart disease (yes/no), myocardial infarction (MI) in the past (yes/no), congestive heart failure (yes/no), stroke (yes/no), obesity (yes/no), and the Charlson Comorbidity Index. However, when constructing a multivariate logistic regression, where candidate confounders were considered as regressors, only gender (m/f), age (years), and arterial hypertension (yes/no) turned out to be statistically significant (Supplementary Materials Table S3). This is largely due to the fact that CKD, CHD, MI, stroke, obesity, and the Charlson Comorbidity Index are closely related to the patient’s age and the existence of arterial hypertension.

After identifying the reliable confounders, the PSM procedure was conducted which enabled two balanced groups with respect to the frequency and distribution of variables: the T2M group remained the same in the number of patients (n = 381), but the control group decreased to 381. The variation ratios for the values of the age and distance confounders (responsible for the presence of Intercept in the logit model) before and after randomization were 0.514/1.067 and 0.938/1.002, respectively. That means that, after pseudo-randomization, the variation ratio became balanced and close to 1. The variables gender and arterial hypertension in the balanced groups became equal in frequency. The distribution by confounding characteristics before and after pseudo-randomization is clearly presented on the eQQ graphs (Supplementary Materials Figure S1). After PSM, the difference in mortality between the balanced control and T2D groups remained significant: in the balanced control group, 362 survivors and 19 deaths (4.99%); and in T2D, 330 and 51 (13.38%), with χ^2^ = 16.108 (*p* < 0.0001).

To stratify the risk of in-hospital death depending on the severity of type 2 diabetes, patients with T2D were divided into two subgroups depending on their blood glucose levels upon admission to COVD-19 hospital: >10 mmol/L (n = 172) and ≤10 mmol/L (n = 209). The use of blood glucose levels for a subgroup analysis was based on the findings of other researchers showing that well-controlled blood glucose with maintenance of glycemic variability between 3.9 and 10.0 mmol/L is associated with a significant reduction in composite adverse outcomes and death [1,16]. In each subgroup, the hospital mortality rate was determined and then compared with the matched (balanced) control group (n = 381). As a result, the rate of hospital mortality in the subgroup with moderate T2D (glucose level ≤10 mmol) was 9.57% (20 cases), which significantly differed from the rate of death in the matched control group (χ^2^ = 4.591, *p* = 0.033). The death rate in the group with severe T2M with a glucose level >10 mmol was even more pronounced, 29.65% (31 cases); thus, it was significantly different with the matched control group at *p* < 0.001 (χ^2^ = 24.488) Also, the mortality rate in severe T2D was higher than that in patients with moderate T2D (χ^2^ = 5.64, *p* = 0.018).

### 3.3. Outcomes in 10 Months Follow-Up

During the 10-month follow-up, some patients required hospital readmission. The causes for hospital readmission included heart failure (61,3%), arrythmias (6.7%), myocardial infarction (1%), stroke (0.6%), pulmonary embolism (0.2%), bleeding (0.1%), and non-cardiovascular diseases (21.1%), and 2.2% died. The median age of deceased subjects at the 10-month FU was 70 [65; 73] years; thus, they were older than the median age of 58 [57; 58] years among survivors, *p* < 0.0001 (Table 4). As expected, T2D patients experienced more strokes compared to controls (1.49% vs. 0.44%, *p* = 0.033). To remove the bias in age, gender, arterial hypertension, CKD, CHD, etc., PSM was conducted to balance the groups. As a result, the difference in stroke frequency decreased from *p* = 0.042 to *p* = 0.090, but the frequency of cardiovascular hospitalizations increased from *p* = 0.942 to *p* = 0.023.

## 4. Discussion

The present study analyzed clinical characteristics, laboratory parameters, and cardiovascular events in the hospital and at a 10-month follow-up in 2463 hospitalized COVID-19 patients, as well as death, risk factors, and changes in laboratory parameters in 381 T2D patients hospitalized with COVID-19-associated pneumonia.

Due to an increase in the prevalence of diabetes with age, patients with T2D infected with SARS-CoV-2 were older than those without diabetes, as expected. Age remains a significant predictor of death in severe and comorbid COVID-19. Patients with T2D were admitted more often in severe condition and with more severe symptoms of COVID-19. Also, T2D patients were characterized by more pronounced respiratory failure and the need for oxygen and lung ventilation (NIVL and MLV) compared with patients without diabetes. These results are consistent with the results of other researchers who have shown that patients with COVID-19 and diabetes are in greater need of hospitalization and transfer to the intensive care unit, non-invasive oxygen therapy, and mechanical ventilation [17,18,19].

These findings can be explained by the changes in the immune system and renin–angiotensin–aldosterone system, together with inflammation, oxidative stress, and endothelial dysfunction in diabetes which have the potential to exaggerate the response triggered by SARS-CoV-2 driving one or more of the cellular processes that result in pulmonary thrombosis, increased vascular permeability and/or cytokine storm, resulting in respiratory failure [20]. The comorbidity index of patients with T2D was four times higher than that of patients without T2D. Comorbidities such as arterial hypertension, CHD, chronic heart failure, obesity, CKD, MI, and stroke were significantly more present in patients with T2D prior to COVID-19 hospitalization, thus supporting previous studies [21].

T2D correlated with worse COVID-19 outcomes: In patients with T2D, the in-hospital mortality was almost three times higher than in patients without the condition, consistent with findings from other researchers [3,7,14,22,23,24,25,26]. Taking into account the disproportions in age, arterial hypertension, CKD, etc., in the groups and between the groups, a PSM procedure was conducted to remove this bias. As a result, the difference in mortality remained highly significant (*p* < 0.0001).

Individuals with T2D often had some degree of chronic inflammation, which may predispose them to cytokine storms and fatal COVID-19. Patients with T2D had significantly increased procalcitonin and CRP compared to individuals without it, as well as neutrophils and ESR. Furthermore, our findings aligned with other preceding research and found that COVID-19 subjects with T2D had greater D-dimer values than those who did not have T2D, likely indicating hemostatic system overactivation. Hyperactivation of the coagulation cascade in COVID-19 in the context of a preexisting pro-thrombotic hypercoagulable state exacerbated by the simple presence of T2D may result in severe thromboembolic outcomes and eventual mortality [21].

The decrease in kidney function, characterized by an increase in the level of creatinine, GRF, and urea and a decrease in serum albumin, was associated with a significantly increased risk of in-hospital death in T2D complicated by COVID-19. These results are supported by other studies evaluating COVID-19 in T2D patients [27]. Recent studies show that COVID-19 is often complicated with acute kidney failure, which is closely associated with higher mortality and morbidity and is an indicator of survival in coronavirus infection [28,29].

It was shown that severe T2D patients had high hospital and FU complication rates in COVID-19 [1]. To stratify the risk of in-hospital death depending on the severity of type 2 diabetes, patients with T2D were divided into two subgroups depending on blood glucose level upon admission to COVD-19-hospital with a cutoff 10 mmol/L, according to the studies of Zhu L. et al. (2020) and Andreeva A.V. et al. (2021) [1,16]. These previous researchers found that well-controlled blood glucose ranging from 3.9 to 10.0 mmol/L is associated with a significant reduction in composite adverse outcomes and death [1,16]. In our study, moderate T2D with glucose level <10 mmol/L had a lower hospital mortality rate than severe (*p* = 0.018), and both T2D groups had higher mortality than control COVID-19 patients (*p* = 0.033 and *p* < 0.001 consequently), which supports the correlation of T2D severity and hospital complication rate.

The study of laboratory parameters of deceased T2D patients showed significant differences compared to survivals. According to a comparative analysis in patients with a fatal outcome, loss of albumin was observed with a decrease in the glomerular filtration rate of more than 1.5 times, along with an increase in the level of urea. The established increase in the intracellular enzyme levels, such as creatine phosphokinase and AST, indicates multiple organ damage caused by the coronavirus infection [30] and/or drug toxicity [31].

The decrease in the absolute number of lymphocytes and the increase in the absolute number of neutrophils in the deceased T2D group compared with discharged T2D patients are associated with progressive macrophage activation syndrome. Despite ongoing anticoagulant therapy, patients with a fatal outcome showed pronounced signs of activation of the hemostatic system, characterized by an increase in D-dimer, thrombocytopenia, and worse control of coagulopathy in this group.

The follow-up of patients over 10 months showed no statistically significant differences between patients with and without T2D, except for stroke (*p* = 0.042). After PSM, the difference decreased in stroke (*p* = 0.090) but increased in cardiovascular hospitalizations (*p* = 0.023). COVID-19 greatly increased the rate of atrial fibrillation during the post-COVID-19 period [32], and T2D is a significant risk factor for the onset of atrial fibrillation [33].

The mechanisms of cerebrovascular manifestations in people with COVID-19 are likely multifactorial. They could be related to conventional stroke mechanisms, with COVID-19 acting as a trigger. Alternatively, they could be directly caused by SARS-CoV-2 infection through specific pathophysiological mechanisms, leading to both ischemic and hemorrhagic stroke.

In both diabetic and non-diabetic patients, readmission rates for cardiovascular and non-cardiovascular conditions, as well as rates of death from various causes, were about the same.

## 5. Conclusions

The in-hospital mortality rates in T2D patients were significantly higher than those in the control group (13.89 vs. 4.89%). The logistic regression analysis showed that the most significant mortality predictors in T2D patients were a Charlson Comorbidity Index > 4, CRP > 66 mg/L, blood glucose > 8.82 mmol/L, and neutrophils > 6.05 × 10^−9^/L. Also increased urea, enzymes (AST, CK), D-dimers, procalcitonin, ferritin, and IL-6; and decreased GFR, albumin, lymphocytes, and platelets are markers with unfavorable prognosis for COVID-19 in T2D patients during the treatment period. T2D patients with a higher glucose level at the time of admission had a higher hospital mortality rate.

With respect to endpoints during the 10-month follow-up, there was a trend toward the increased non-cardiovascular hospitalizations, myocardial infarction, pulmonary embolism (*p* > 0.05); except for stroke and cardiovascular hospitalizations, which was significantly higher in T2D group.

### Limitations of the Study

This study was a single-center study. The treatment of COVID-19 changed during 2020–2021 according to current guidelines, which may have impacted patient survival rates. HbA1c is a gold-standard of T2D severity; however, it was recorded in just a small number of our T2D patients.

## Figures and Tables

**Table 1:** Demographics, clinical characteristics, and laboratory parameters upon admission of patients hospitalized with COVID-19.

Parameter	Control COVID-19, N = 2082 Median (Q1; Q3) or n (%)	COVID-19 + T2D, N = 381 Median (Q1; Q3) or n (%)	*p*
Gender, m/f	930 (44.67%)/1152 (55.33%)	126 (33.07%)/255 (66.93%)	<0.0001
Age, years	57 [56; 58]	66 [65; 68]	<0.0001
Hospitalization, days	11 [11; 11]	11 [11; 12]	0.0018
Duration of disease, days	15 [15; 15]	16 [15; 16]	0.0008
Outcome (survivals/deceased)	1980 (95.1%)/102 (4.89%)	330 (86.61%)/51 (13.39%)	<0.0001
COVID-19 related symptoms upon admission:			
Dyspnea at rest, n (%)	682 (32.87%)	121 (31.75%)	0.7032
Dyspnea at exertion, n (%)	391 (18.84%)	252 (66.14%)	<0.0001
Incidences of aches, n (%)	1372 (66.15%)	236 (61.94%)	0.1360
Weakness, n (%)	1992 (96.05%)	362 (95.01%)	0.5632
Events in hospital			
RDS, n (%)	252 (12.32%)	43 (11.29%)	0.6401
Lung tissue damage on CT, %	40% [36; 40]	40% [40; 44]	0.0015
O_2_-dependent, n (%)	104 (5.02%)	29 (7.61%)	0.0383
Non-invasive ventilation, n (%)	28 (1.35%)	15 (4.55%)	<0.0001 ^ǂ^
Mechanical intubation, n (%)	81 (3.9%)	35 (9.19%)	<0.0001
Relevant concomitant diseases			
Arterial hypertension, n (%)	769 (36.97%)	306 (80.31%)	<0.0001
Chronic kidney disease, n (%)	44 (2.11%)	60 (15,75%)	<0.0001
Coronary heart disease, n (%)	195 (9.37%)	86 (22.57%)	<0.0001
MI in the past, n (%)	58 (2.79%)	35 (9.19%)	<0.0001
Congestive heart failure, n (%)	176 (8.46%)	73 (19.16%)	<0.0001
Stroke, n (%)	46 (2.21%)	20 (5.25%)	0.0002 ^ǂ^
Valves replacement, n (%)	6 (0.29%)	4 (1.05%)	0.0870 ^ǂ^
COPD, n (%)	73 (3.51%)	6 (1.58 %)	0.0712 ^ǂ^
Obesity, n (%)	36 (1.73%)	18 (4.72%)	<0.0001 ^ǂ^
The Charlson Comorbidity Index	1 [1; 1]	4 [4; 4]	<0.0001
Laboratory parameters:			
Hematocrit, %	39.6 [39.4; 39.8]	38.65 [37.9; 39.31]	0.0024
Hemoglobin, g/L	133 [132; 133.6]	130 [128; 132]	0.0098
Neutrophils, ×10^9^/L	4.01 [3.88; 4.19]	4.33 [3.97; 4.69]	0.0252
Lymphocytes, ×10^9^/L	1.11 [1.09; 1.15]	1.08 [0.98; 1.17]	0.1374
Monocytes, ×10^9^/L	0.3425 [0.3140; 0.3640]	0.3595 [0.3040; 0.40]	0.6017
Platelets, ×10^9^/L	195 [192; 201]	191.5 [183; 205]	0.5707
ESR, mm/h	30 [29; 31]	35 [32; 37]	<0.0001
CRP, mg/L	24 [22.0; 26]	42 [35; 49.5]	<0.0001
Procalcitonin, ng/L	0.09 [0.09; 0.1]	0.14 [0.12; 0.17]	<0.0001
Total protein, g/L	41.9 [41.64; 42.2]	41.5 [40.8; 42.4]	0.9113
Albumin, g/L	40.9 [40.6; 41.2]	40.4 [39.7; 41.0]	0.0160
Total bilirubin, mcmol/L	8.1 [8.0; 8.3]	8.0 [7.7; 8.7]	0.4598
AST, U/L	28.6 [27.8; 29.6]	28.1 [26.3; 29.9]	0.2215
ALT, U/L	29.4 [28.6; 30.7]	28.9 [27.1; 30.57]	0.5213
Total iron, mcmol/L	8.7 [8.2; 9.1]	7.7 [6.7; 8.8]	0.0023
Ferritin, ng/mL	370.39 [339; 402.7]	380.99 [327.51; 432.43]	0.6367
Glucose, mmol/L	6.18 [6.1; 6.3]	9.46 [8.82; 10.30]	<0.0001
Lactate, mmol/L	3,1 [3.0; 3.2]	3.0 [2.7; 3.2]	0.0602
CK, U/L	108 [102; 113]	113.5 [97.43; 134.87]	0.2067
Alkaline phosphatase, U/L	165.05 [162.7; 167.54]	171.3 [162.73; 179.55]	0.1537
Urea, mmol/L	5.39 [5.29; 5.5]	6.95 [6.59; 7.35]	<0.0001
GFR, ml/min/m^2^	71 [70; 71]	62 [61; 64]	<0.0001
D-Dimer, ng/mL	222 [212; 236.79]	317 [270.47; 362.07]	<0.0001
INR	1.02 [1.01; 1.02]	1.05 [1.03; 1.06]	0.0003
PTT, sec	13.8 [13.7; 13.9]	14.0 [13.8; 14.1]	0.1797
Sodium, mmol/L	143 [143; 143]	142 [141; 142]	<0.0001
Potassium, mmol/L	4.2 [4.1; 4.2]	4.3 [4.2; 4.3]	0.0003
IL-6, pg/mL	6.18 [4.94; 7.89]	8.46 [5.08; 14.27]	0.0907

P.s.: ^ǂ^ Chi-quadrate text with Yates correction; ALT—alanine aminotransferase; AST—aspartate aminotransferase; CK—creatine kinase; COPD—chronic obstructive pulmonary disease; CRP—C-reactive protein; CT—computer tomography; ESR—erythrocytes sedimentation rate; GFR—glomerular filtration rate; IL-6—interleukin-6; INR—international normalization ratio; MI—myocardial infarction; PTT—partial thromboplastin; RDS—respiratory distress syndrome; T2D—type 2 diabetes mellitus.

**Table 2:** The results of the ROC analysis of risk factors of hospital mortality.

Predictors	Cutoff Point	Sens./Specif., %	*p*-Value of ROC
CCI	>4	80.0/62.5	<0.0001
Glucose	>8.82 mmol/L	74.0/48.1	0.0142
Neutrophils	>6.05 × 10^9^/L	67.5/70.2	0.0001
CRP	>66 mg/L	61.2/67.1	0.0008

P.s.: CCI—Charlson Comorbidity Index; CRP—C-reactive protein.

**Table 3:** The results of multivariant logistic regression analysis of hospital mortality.

Predictors	β-Coefficient ± SE	OR	CI OR 95%	*p*-Value of β
CCI	1.9148 ± 0.4375	6.79	4.38–10.51	<0.0001
Glucose	0.9643 ± 0.4469	2.62	1.68–4.10	0.0310
Neutrophils	1.5141 ± 0.3918	4.55	3.07–6.73	0.0001
CRP	0.8945 ± 0.3842	2.45	1.67–3.59	0.0199
Free variable	−4.9042 ± 0.6058			<0.0001

P.s.: CCI—Charlson Comorbidity Index; CRP—C-reactive protein; OR—odds ratio; CI OR 95%—95% confidence interval.

**Table 4:** Cardiovascular endpoints in 10-month follow-up after hospitalization.

Parameters	Control COVID-19	COVID-19 + T2D	Control COVID-19, Matched	p_1_	p_2_
n (%)	1922	269	362		
Non-cardiovascular hospitalizations	408 (21.23%)	67 (24.9%)	70 (19.34%)	0.171	0.094
Cardiovascular hospitalizations	98 (5.09%)	14 (5.20%)	37 (10.22%)	0.942	0.023
Bleeding	2 (0.1%)	1 (0.37%)	1 (0.28%)	0.817 ^ǂ^	0.614 ^ǂ^
Myocardial infarction	21 (1.09%)	2 (0.74%)	7 (1.93%)	0.837 ^ǂ^	0.365 ^ǂ^
Stroke	9 (0.47%)	4 (1.49%)	1 (0.28%)	0.042	0.090
Pulmonary embolism	4 (0.21%)	1 (0.37%)	1 (0.28%)	0.877 ^ǂ^	0.614 ^ǂ^
Death	41 (2.13%)	9 (3.35%)	11 (3.04%)	0.212	0.828
Combined endpoint (cardiovascular hospitalization + stroke + myocardial infarction + pulmonary embolism + death)	161 (8.38%)	27 (10.04%)	46 (12.71%)	0.363	0.300

^ǂ^ Chi-quadrate test was performed with Yates correction; p_1_—p-level between controls (n = 1922) and T2D patients (n = 269); p_2_—p-level between controls matched (n = 362) and T2D patients (n = 269).

## Supplementary Materials

The following supporting information can be downloaded at https://www.mdpi.com/article/10.3390/biomedicines12020467/s1, Table S1: Comparison of discharged and deceased hospitalized patients with COVID-19-associated pneumonia; Table S2: Univariant analysis of mortality risk factors for hospitalized patients with COVID-19-associated pneumonia; Table S3: Results of the logit model estimation for assessing the reliability of confounders; Figure S1: eQQ Plots confounding characteristics distribution for “arterial hypertension” confounder.
