# Supplementary material for: Long-Term Outcomes of COVID-19 in Hospitalized Type 2 Diabetes Mellitus Patients
Source: Biomedicines. 2024 Feb 19;12(2):467. doi: 10.3390/biomedicines12020467 (PMC10886829; doi:10.3390/biomedicines12020467)
Supplement: Supplementary file 1 [file biomedicines-12-00467-s001.zip › biomedicines-2851055-supplementary.pdf]

Supplementary Table S1. Comparison of discharged and deceased hospitalized patients with COVID-19-associated pneumonia and T2D

Parameter	Discharged, n=330 Median (Q1; Q3) or n (%)	Deceased, n=51 Median (Q1; Q3) or n (%)	P
Gender, m/f	110 (33.33%) / 220 (66.67%)	16 (31.37%) / 35 (68.63%)	꩜꩜꩜꩜꩜꩜
Age, years	65 [64; 67]	71 [69;74]	**⚜0.0001**
Duration of disease, days	12 [11; 12]	10,5 [6; 14]	0.1094
Outcomes (survivals/deceased)	16 [15; 16]	15 [12; 22]	0.9644
COVID-19 related symptoms at admission:			
Severity (moderate/severe)	288 (87.27%)/42 (12.73%)	10 (25.49%)/41 (74.51%)	**< 0.0001**
Dyspnea at rest	108 (32.72%)	13 (25.49%)	0.3024
Dyspnea at exertion	214 (64.85%)	38 (74.51%)	0.1749
Incidences of aches	196 (59.39%)	40 (78.43%)	**0.0101**
Weakness	312 (94.55%)	50 (98.04%)	0.4714ǂ
Events in hospital			
RDS	3 (0.91%)	40 (78.43%)	**⚜0.0001**
Lung tissue damage on CT, %	40 [40; 44]	49 [40; 60]	0.0660
O_2-dependent	12 (3.64%)	17 (33.33%)	**⚜0.0001**
Noninvasive ventilation	4 (1.21%)	10 (19.61%)	**⚜0.0001**
Mechanical intubation	5 (1.52%)	30 (58.82%)	**⚜0.0001**
Relevant concomitant diseases			
Arterial hypertension	264 (80.0%)	42 (82.35%)	0.6952
Chronic kidney disease	47 (14.24%)	13 (25.49%)	**0.0413**
Coronary heart disease	70 (21.21%)	16 (31.37%)	0.1069
MI in the past	31 (9.39%)	4 (7.84%)	0.7222
Congestive heart failure	62 (18.79%)	11 (21.57%)	0.6391
Stroke	17 (5.15%)	3 (5.88%)	0.8282
Valve replacement	3 (0.91%)	1 (1.96%)	0.4928
COPD	5 (1.52%)	1 (1.96%)	0.8119
Obesity	13 (3.94%)	5 (9.80%)	0.0670
The Charlson comorbidity index	4 [4; 4]	6 [5; 7]	0.0001
Laboratory parameters:			
Hematocrit, %	38.4 [37.5; 39.38]	39.4 [38.1; 41.66]	0.3208
Hemoglobin, g/l	130 [128; 132]	134 [124; 139.7]	0.2593
Neutrophils, *10^9/l	4.25 [3.85; 4.5]	8.4 [6.21; 12.29]	**⚜0,0001**
Lymphocytes, *10^9/l	1.11 [0.99; 1.2]	0.924 [0.76; 1.13]	**0.0363**
Monocytes, *10^9/l	0.3455 [0.3; 0.4]	0.3975 [0.34;0.47]	0.3932
Platelets, *10^9/l	197 [186; 210]	162 [144.34; 191.66]	**0.0161**
ESR, mm/h	35 [31; 37]	39 [31,34; 46,66]	0.2391
CRP, mg/l	39 [39.93; 48]	75.5 [45.61; 83.25]	**0.0012**
Procalcitonin, ng/l	0.13 [0.11; 0.15]	0.67 [0.19; 1.3]	**0.0001**
Albumin, g/l	40.7 [40.12; 41.18]	38 [36.85; 39.67]	**0.0031**
Total bilirubin, mcmol/l	7.9 [7.5; 8.5]	9.3 [7.6; 11.8]	0,1161
AST, U/l	26.8 [24.8; 28.8]	40.95 [32.6; 44.98]	**⚜0.0001**
ALT, U/l	28.2 [26.32; 29.48]	26.9 [24.06; 32.94]	0.5266
Ferritin, ng/mL	354.06 [306.9; 419.3]	500 [416.6; 500]	**0.0197**

Glucose, mmol/l	9.0 [8.4; 9.9]	11.55 [9.62; 14.43]	**0.0102**
Lactate, mmol/l	3.05 [2.8; 3.3]	2.55 [1.8; 2.9]	0,0753
CK, U/l	105 [93.6; 123]	174 [119.4; 266.9]	**0,0352**
Alkaline phosphatase, U/l	171.7 [163.3; 182.1]	160.5 [139.8; 188.7]	0.4048
Urea, mmol/l	6.79 [6.4; 7.2]	9.3 [7.5; 10.9]	**0.0001**
GFR, ml/min/m^2	63 [61.5; 66]	52 [43.3; 59.8]	**0.0004**
D-Dimer, ng/ml	308[259.8; 356]	513 [216.6; 1130.9]	**0.0587**
INR	1.04 [1.03; 1.06]	1.06 [1.02; 1.09]	0.3705
PTT, sec	13.9 [13.7; 14.1]	14.4[13.7; 14.7]	0.1607
Sodium, mmol/l	142 [141; 142]	141 [139.8; 142]	**0.0146**
Potassium, mmol/l	4.3 [4.2; 4.3]	4.2 [4.0; 4.5]	0,8962
IL-6, pg/mL	8.08 [4.26; 11.85]	27.06 [6.43; 187.35]	**0.0086**

[1] P.s.: ǂ - Chi-quadrate text with Yates correction, ALT – alanine aminotransferase, AST – aspartate aminotransferase, CK – creatine kinase, COPD - chronic obstructive pulmonary disease, CRP - C-reactive protein, CT - computer tomography, ESR – erythrocytes sedimentation rate, GFR - glomerular filtration rate, IL-6 – interleukin-6, INR – international normalization ratio, MI – myocardial infarction, PTT – partial thromboplastin, RDS - respiratory distress syndrome, T2D - type 2 diabetes mellitus.

Supplementary Table S2. Univariant analysis of mortality risk factors in hospitalized patients with COVID-19-associated pneumonia and T2D

Indicator	Coefficient	STD. Error	ODDS RATIO	95% CI	**P**
Age	0,07978	0,01709	1,0831	1,0474; 1,1199	**<0,0001**
Condition	3,0285	0,4064	20,6667	9,3187;45,8339	**<0,0001**
Albumin, g/l	-0,1261	0,03884	0,8815	0,8479;0,9164	**0,0012**
Urea, mmol/l	0,1177	0,03384	1,1249	1,0875;1,1636	**0,0005**
AST, U/l	0,0093	0,0046	1,0093	1,0047;1,0139	0,0433
The Charlson comorbidity index	0,5775	0,1013	1,7816	1,6099;1,8715	**<0,0001**
Creatine phosphokinase, U/l	0,0005	0,0002	1,0005	1,0003;1,0007	**0,0253**
CRP, mg/l	0,0169	0,0056	1,017	1,0113;1,0228	**0,0024**
Ferritin, ng/ mL	0,0034	0,0016	1,0034	1,0002;1,0050	**0,0387**
Procalcitonin, ng/l	0,1299	0,0572	1,1387	1,0754;1,2058	**0,0232**
GFR, ml/min/m2	-0,03795	0,008945	0,9628	0,9542;0,9714	**<0,0001**
Glucose, mmol/l	0,0960	0,0260	1,1008	1,0725;1,1298	**0,0002**
IL-6, pg/mL	0,0040	0,0022	1,0040	0,9998;1,0083	0,0627
Lymphocytes, *10^9/l	-0,2549	0,3082	0,7750	0,4236;1,4180	0,4083
Neutrophils,*10^9/l	0,1062	0,0322	1,1121	1,0768;1,1484	**0,0010**
Platelets, *10^9/l	-0,0055	0,002544	0,9944	0,9895;0,9994	**0,0285**
Sodium, mmol/l	-0,01236	0,01389	0,9877	0,9612;1,0150	0,3734

P.s.: AST – aspartate aminotransferase, CRP – C-reactive protein, GFR - glomerular filtration rate, IL-6 – interleukin 6.

Supplementary Table S3. Results of the logit model estimation for assessing the reliability of confounders

Confounding variable	β-coefficient± SE	P-value of β
Intercept	-4.7252±0.3348	**<0.0001**
Gender, male	-0.344±0.1246	0.0057
Age, years	0.0377±0.0053	<0.0001

Arterial hypertension	1.4422±0.141	<0.0001

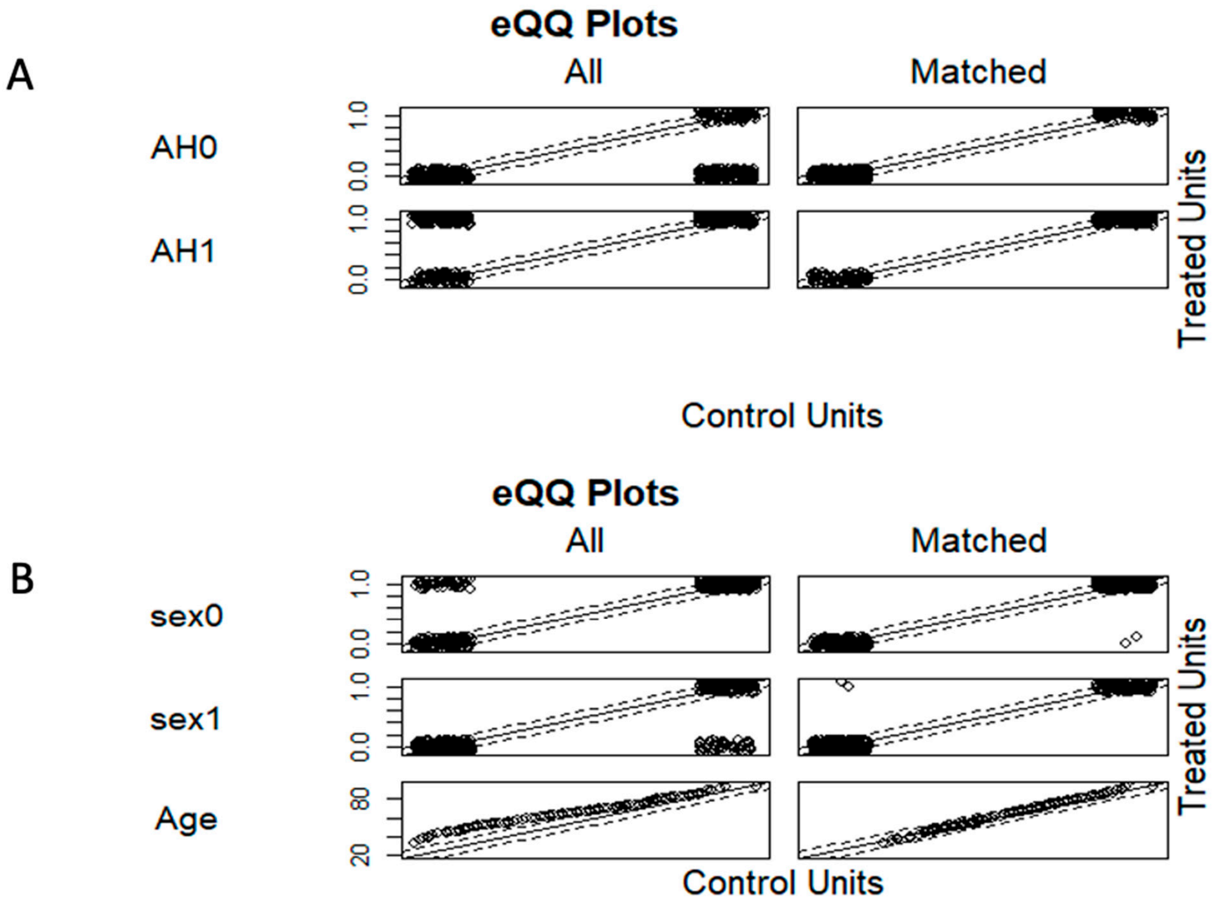

Supplementary Figure S1. eQQ Plots confounding characteristics distribution for “Arterial hypertension” confounder: AH0 – “No”, AH1 – “Yes” (A) and for confounders “Age” and “Gender”: sex0 – “female”, sex1 – “male” (B) before and after pseudo-randomization.
